# The Water Content Drives the Susceptibility of the Lichen *Evernia prunastri* and the Moss *Brachythecium* sp. to High Ozone Concentrations

**DOI:** 10.3390/biology9050090

**Published:** 2020-04-27

**Authors:** Andrea Vannini, Giulia Canali, Mario Pica, Cristina Nali, Stefano Loppi

**Affiliations:** 1 Department of Life Sciences, University of Siena and Italy, 53100 Siena, Italy; andrea.vannini@unisi.it (A.V.); giulia.canali@student.unisi.it (G.C.); 2 Bioredox, 00187 Rome, Italy; bioredoxsrl@gmail.com; 3 Department of Agriculture, Food and Environment, University of Pisa, 56124 Pisa, Italy; cristina.nali@unipi.it

**Keywords:** antioxidants, chlorophyll, cryptogams, hydration state, photosynthesis

## Abstract

The aim of this study was to evaluate the tolerance of lichens (*Evernia prunastri*) and mosses (*Brachythecium* sp.) to short-term (1 h), acute (1 ppm) O_3_ fumigation under different hydration states (dry, <10% water content, metabolism almost inactive; wet, >200% water content, metabolism fully active). We hypothesized that stronger damage would occur following exposure under wet conditions. In addition, we checked for the effect of recovery (1 week) after the exposure. Ozone fumigation negatively affected the content of chlorophyll only in wet samples, but in the moss, such a difference was no longer evident after one week of recovery. Photosynthetic efficiency was always impaired by O_3_ exposure, irrespective of the dry or wet state, and also after one week of recovery, but the effect was much stronger in wet samples. The antioxidant power was increased in wet moss and in dry lichen, while a decrease was found for wet lichens after 1 week. Our results confirm that the tolerance to O_3_ of lichens and mosses may be determined by their low water content, which is the case during the peaks of O_3_ occurring during the Mediterranean summer. The role of antioxidant power as a mechanism of resistance to high O_3_ concentrations needs to be further investigated.

## 1. Introduction

Ozone (O_3_) is a strongly oxidizing pollutant occurring at ground level as a consequence of the interaction between solar irradiation and gases such as nitrogen oxides (NOx), volatile organic compounds (VOCs), and carbon monoxide (CO). Considering its known phytotoxicity [1] and the global tendency of increasing background concentrations of about 0.3 ppb/y as a consequence of the global increases in temperature and precursors [2], investigations of the impact of O_3_ on vegetation have been strongly prompted during the last few years [3], especially because this compound may affect plants’ productivity [4]. 

Lichens, despite their wide use as bioindicators of air quality [5], are known for being rather insensitive to O_3_ pollution, with lab and field studies showing similar results: a (very) limited influence of O_3_ on lichen physiology and biodiversity. In detail, field studies did not find any evidence of a correlation between lichen biodiversity and O_3_ concentrations, as evaluated indirectly by the damage occurring in the O_3_ supersensitive plant species *Nicotiana tabacum* Bel-W3 during summer exposures [6,7,8], while O_3_ fumigations at ecologically relevant concentrations under controlled conditions failed to cause relevant injuries to the photosynthetic systems of several lichen species [9,10,11]. These results suggested a very high tolerance of these organisms to environmental O_3_ concentrations, with a toxicity threshold higher than the natural concentrations to which they are commonly exposed to.

However, upon increasing the concentrations of O_3_ up to levels less or not at all ecologically relevant, the results were different. Fumigations carried out at ca. 1 ppm O_3_ indicated contrasting results: a decrease in net photosynthesis in *Parmelia sulcata* [12] and no change in the chlorophyll content of *Cladonia arbuscula*, the photobiont of *Cladonia stellaris* [13] or the photosynthesis of *Cladonia rangiformis* [14]. However, acute fumigations at 3 ppm O_3_ induced strong physiological and ultrastructural damage in both the photobiont and the mycobiont of the pollution-tolerant species *Xanthoria parietina* [15]. Nevertheless, these authors suggested that the hydration state may play a major role in determining the severity of the damage, thus explaining the ecological insensitivity of lichens to the high environmental levels of O_3_ occurring during dry Mediterranean summers.

Similarly to lichens, a limited susceptibility to O_3_ of bryophytes has been reported. Fumigations with 150 ppb O_3_ for 5 h induced a slight decrease in photosynthesis only in one out of four *Sphagnum* species [16], while fumigations with 70–80 ppb O_3_ for 6–9 weeks induced only a modest photosynthetic injury in *Sphagnum recurvum* compared with in *Polytrichum commune* [17]. In addition, chronic fumigations of *Sphagnum* in open top chambers did not induce reductions in the chlorophyll contents after exposure to 50, 100 and 150 ppb O_3_ [18]. Nevertheless, fumigations carried out for 10 weeks (6 h/d, 4 d/w) with 240 and 320 ppb were shown to cause reductions in the abundance of four mosses species [19].

Poikilohydric organisms like lichens and mosses are assumed to have higher resistance to gaseous pollutants during the dry state, having a metabolism strictly dependent on their water content [20], and since during the summer periods of higher O_3_ concentrations the metabolism of these cryptogams is largely reduced, they are likely to be less prone to being injured [6]. Nevertheless, information about the resistance of mosses to O_3_ exposure during different hydration states is lacking and in need of clarification.

The aim of this study was to evaluate the tolerance of lichens and mosses to short-term acute O_3_ fumigation under different hydration states. We hypothesized that stronger damage would occur following exposure under wet conditions. In addition, we checked for the effect of recovery after exposure.

## 2. Materials and Methods

### 2.1. Experimental

Samples of the lichen *Evernia prunastri* and the moss *Brachythecium* sp. were collected at the end of September 2019 in a remote area of the Siena province (Tuscany, Central Italy), far from any local source of pollution. These species were chosen considering their wide use as biological indicators as well as their ease of collection and handling. In the laboratory, samples were cleaned from extraneous material under a stereoscopic microscope using plastic tweezers. After cleaning, one batch of 18 samples was air-dried (residual water <10%) overnight in a climatic chamber at 16 °C and 55% relative humidity (RH), while another batch of 18 samples was fully hydrated (water content up to 250% of dry weight for the lichen, and up to 600% for the moss) overnight in a climatic chamber at 16 °C and 90% RH. Two thirds of the air-dried and fully hydrated samples were fumigated for 1 h at an ozone concentration of 1 ppm using an ozone generator (GPC2000, Ozonosoluzioni, Italy); the remaining third were fumigated for 1 h with O_3_-free air (control samples). To evaluate the possible recovery of the samples, 50% of the fumigated and control samples were left for 1 week under environmental conditions. 

### 2.2. Physiological Parameters

#### 2.2.1. Chlorophyll Content

The total chlorophyll content of the lichen and moss samples was measured using a chlorophyll-content meter (CCM-300, Opti-Science, Hudson, NY, USA), which indicates the chlorophyll content on a surface basis (mg/m^2^). The effectiveness of this non-destructive method for the analysis of the chlorophyll content has already been tested [21]. Ten replicates were measured for each experimental unit.

#### 2.2.2. Chlorophyll Fluorescence Analysis

The analysis of the chlorophyll a fluorescence of the photosystem II and the analysis of the chlorophyll fluorescence transient (OJIP) are known reliable techniques to assess the performance of photosynthetic organisms following O_3_ exposures [22]. In this study, the chlorophyll a fluorescence was investigated using its most common parameter F_V_/F_M_, which indicates the maximum quantum efficiency of PS II photochemistry, an indicator of photosynthetic efficiency. The analysis of the chlorophyll a fluorescent transient (OJIP test) was used as a supplementary photosynthetic indicator. Prior to analysis, samples were hydrated in a climatic chamber at 16 °C, 90% RH and 40 μmol/m^2^/s photosynthetically active radiation (PAR), to obtain their maximal photosynthetic activation. The analysis was run, lighting the samples for 1 sec with a saturating 3000 μmol/m^2^/s red light pulse, using a Plant Efficiency Analyzer (Handy PEA, Hansatech Ltd, Norfolk, UK). Fifteen replicates were measured for each experimental unit.

#### 2.2.3. Total Antioxidant Power

The DPPH assay is a simple and functional method to evaluate the response of the total antioxidant activity after O_3_ exposure [23]. Samples of ca. 50 mg were homogenized in 1 mL of a solution of ethanol/water (80:20; *v*/*v*). Of the homogenate, 100 µL were added to 1 mL of a 100 µM DPPH solution prepared by dissolving 3.9 mg of this compound in 100 mL of methanol/water (80:20; *v*/*v*). After the reaction, which occurred in 1 h, samples were read at 517 nm and the results were expressed as % Antiradical Activity (ARA%) according to the formula:

ARA% = 100 × [1 − (control absorbance/sample absorbance)](1)

where control absorbance = the absorbance of the reagents only. Five replicates were measured for each experimental unit.

### 2.3. Statistical Analysis

Results were expressed as ratios to control samples. Owing to the limited data set, non-parametric statistics were used. Outliers were sought for using the Tukey test. Differences between fumigated and control samples were checked with the Mann-Withney U test. Differences between dry and wet samples, both immediately after fumigation and after recovery, were checked using the non-parametric Kruskal–Wallis ANOVA. The Dunn’s test was used for post-hoc comparisons, except for temporal ones, for which the Wilcoxon rank sum test was used. In both cases, a correction for multiple testing was applied according to [24]. All calculations were run using the free software R [25].

## 3. Results

The physiological parameters in *Evernia* sp. and *Brachythecium* sp. immediately after O_3_ fumigation, as well as after one week from the latter, are summarized in Table 1.

Compared with control samples, 1 h of fumigation with 1 ppm O_3_ negatively affected the content of chlorophyll only in wet samples, but in moss samples, such a difference was no longer evident after one week of recovery. Photosynthetic efficiency was always impaired by O_3_ exposure, irrespective of dry or wet conditions, and also after 1 week of recovery, but the effect was much stronger for wet samples. The antioxidant power was increased in wet moss and in dry lichen, while a decrease was found for wet lichens after 1 week.

Differences between dry and wet samples emerged for all the investigated parameters, with wet samples generally showing lower values.

After one week of recovery, moss wet samples showed an almost complete restoration of their chlorophyll content. The photosynthetic efficiency of dry samples almost doubled in moss but did not change in lichens. The antioxidant power decreased in both lichens and mosses.

**Figure 1:**
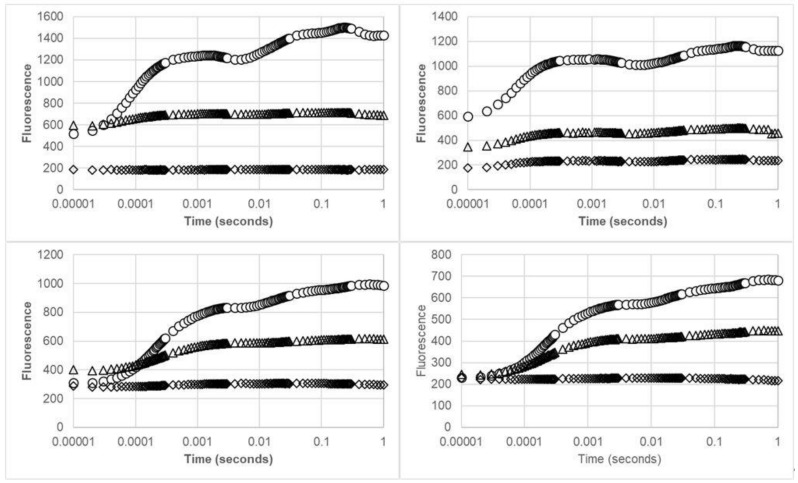
OJIP fluorescence transients of the lichen *Evernia prunastri* (up) and the moss *Brachythecium sp.* (down) after the O_3_ fumigation (left) and the recovery time (right). Legend: (○) control samples (mean of dry and wet), (∆) dry fumigated samples, (◊) wet fumigated samples.

The fluorescence transient curves (Figure 1) confirm that remarkable injury occurred to the photobiont of both organisms following O_3_ fumigations, which persisted after 1 week of recovery. The transient curves flattened out, losing their typical sequence of the OJIP steps as indicated for healthy (control) samples. Fluorescence emission was characterized by a marked reduction in F_M_ values in dry fumigated samples of both organisms, while negligible differences between F_0_ and F_M_ values were observed for wet fumigated samples, which showed almost flat transients.

## 4. Discussion

Short-term acute fumigation with 1 ppm O_3_ impaired the photosynthetic efficiency of the lichen, with effects being much more evident in the hydrated state and with no recovery after the damage. However, a decreased chlorophyll content was found only in wet samples, suggesting that the mechanisms of action of O_3_ causing these alterations are not identical. The antioxidant power always being higher in dry samples suggests that a counteraction of the strong oxidizing effect of O_3_ may be a key factor to protect the integrity of the chlorophyll. Nevertheless, there is clear evidence that O_3_ sensitivity is dependent upon the hydration state, since—also in terms of photosynthetic efficiency—the damage was much more striking in wet samples, of which the capacity to convert light was almost abolished. The response was similar for the moss, which was, however, less sensitive to O_3_ stress and showed clear recovery after one week from the fumigation, even if full recovery was achieved only for the chlorophyll content. Unlike the lichen, the moss showed higher values of antioxidant power in wet samples, thus suggesting a role of this latter parameter in protecting the photosynthetic system.

The higher impact of O_3_ on wet samples may be determined by the combination of two main factors, the great solubility of this pollutant in water and the fully activated metabolism of both organisms during the hydrated state. Ozone is a very soluble molecule that exhibits a water solubility higher than that of oxygen and a lower affinity for the cellular hydrophobic layers [26]. After its dissolution in water, O_3_ starts its decomposition, leading to the formation of reactive oxygen species (ROS), responsible for all of the oxidizing reactions that further occurred [27]. Despite ROS being (at low concentrations) natural components of cell metabolism, produced as a result of aerobic respiration and used in several cell processes, exposure to O_3_ tends to increase their concentration, generating cellular perturbations, changes in cellular homeostasis and further physiological injuries, following a process known as “oxidative burst” [28]. The increased metabolism of these cryptogamic species during the hydrated state may have increased their susceptibility to O_3_. In fact, being poikilohydric organisms, with metabolic activity largely dependent on the hydration state, lichens and mosses tend to increase their net photosynthetic rate (NPR) under increasing hydration conditions [29,30,31,32]. However, the combination of these two factors—the O_3_ dissolution in water and the activated metabolism during the hydrated state—are probably the main reasons explaining the differential toxicity of lichens and mosses to this pollutant. This behavior, dependent on the hydration state, is known also for other toxic gaseous pollutants such as SO_2_, which shows a higher toxicity to lichens and mosses when they are completely wet [33,34], in spite of their activated metabolisms and the high affinity of SO_2_ for water [35].

The mechanism of action of O_3_ against the chlorophyll pool is complex [26], but it is generally assumed to be related either to the direct action of ROS on chlorophylls, with subsequent degradation (chlorosis), or to a sort of protection mechanism of the photosynthetic system [36]. The reduction in the chlorophyll content of wet samples of *E. prunastri* after O_3_ fumigation is at variance with the results of [15], which showed a reduction in dry samples of the lichen *Xanthoria parietina*, probably determined by the absence of water, which allowed easy O_3_ intrusion into the lichen thallus, as noted for CO_2_, for which water has an active role in limiting its diffusion inside the lichen cortex [29,37]. Lichens may show remarkable differences in O_3_ uptake during the dry state [38], probably depending on differences in their morphology [39] or tissue structure—as also suggested for vascular plants [26]—or on other (still unclear) physiological characteristics that also drive their resistance to others pollutants.

The ecologies of *Evernia* and *Xanthoria* is quite different, the former being hygrophytic and the latter, xerophytic [40]; thus, they are naturally less hydrated, with a consequent increase in their tolerance to pollutants such as SO_2_ [34,41]. In spite of their differential ecophysiologies, *Evernia* is known to be more hydrophobic than *Xanthoria* [42,43], the latter having the capacity to hydrate up to 300% of its dry weight vs. the 200% for the former. In addition, once fully hydrated, to achieve full evaporation, *Evernia* requires up to 60 min, while *Xanthoria* may prolong the hydrated state for a much longer time, up to 180 min [43]. These marked differences may well explain the different responses of these two species to O_3_.

The limited chlorophyll reduction in the wet samples of *Brachythecium* is consistent with chronic fumigations (4–6 weeks) carried out on *Sphagnum* in open top chambers, during the dry (Summer, RH = 52–80%) and the wet (Autumn, RH = 71–92%) seasons, for which significant reductions in the chlorophyll contents were not found after exposure to a wide range (50–150 ppb) of O_3_ concentrations [18]. These results suggest a limited effect of O_3_ on chlorophyll when samples are not under fully hydrated conditions. However, reduction in the chlorophyll content as a consequence of the toxic action of O_3_ has been reported for several vascular plants when fumigated under different concentrations, e.g., for soybean (*Glycine max*) at 50–130 ppb O_3_ [44], wheat (*Triticum aestivum*) at 25–35 ppb O_3_ [45], bean (*Phaseolus vulgaris*) at 50–90 ppb O_3_ [46], and *Tilia americana* at 120 ppb O_3_ [47].

The acute O_3_ fumigation severely impaired the photosynthetic efficiency of both the lichen and the moss, with reductions of ca. 100% in wet samples and ca. 65–80% in dry samples. Similar results were observed for the lichen *X. parietina*, for which wet and dry samples showed reductions of ca. 85% and 69%, respectively [15]. Slight reductions in the photosynthetic efficiency of *Evernia* were observed after 6 weeks of exposure to 90 ppb O_3_ in a partially continuous hydration state [38], but were not observed when fumigated for 14 days with 300 ppb of O_3_ (4 h/d) under high levels of humidity (RH = 97%) [48]. The fumigations of four species of *Sphangnum* at 150 ppb O_3_ for 5 h induced slightly decreased photosynthetic efficiency in one species only [16], while fumigations of *Sphagnum recurvum* and *Polytrichum commune* with 70–80 ppb O_3_ for 6–9 weeks induced the occurrence of modest photosynthetic injuries only in the former [17]. The effect of O_3_ fumigations on the photosynthetic efficiency of higher plants is well documented, and significant effects were reported in ca. 50% of the studies [49], e.g., in the summer squash (*Cucurbita pepo*), which showed an impairment of this parameter when fumigated for 5 h/d for 5 days with 150 ppb O_3_ [36]. Furthermore, reductions were also observed in *Tilia americana* after 28–42 days of exposure to 120 ppb O_3_ at 5 h/day [47], in sugar beet (*Beta vulgaris*) and spring rape (*Brassica napus*) at 35 ppb O_3_ [50], and wheat (*Triticum aestivum*) at 50 ppb O_3_ at different growth stages [51].

After 1 week following fumigation, the dry samples of *Brachythecium* showed an almost complete recovery of the chlorophyll content and an increase in photosynthetic efficiency. The occurrence of photosynthetic recovery after O_3_ exposure was also observed in some vascular plants after restoration in O_3_-free air. In detail, *Solanum tuberosum* showed a recovery of its net photosynthesis after 4 days of fumigation at 60–80 ppb O_3_ [52], while *Nicotiana tabacum* Bel B (ozone tolerant) did so after 17 h following fumigation with 300 ppb [53]. In addition, *Quercus ilex* and *Q. pubescens* showed recoveries in their photosynthesis after 72 h following 4 days of exposure to 9 h/d 300 ppb O_3_ [54].

The analysis of the chlorophyll fluorescence transient confirmed the strong negative effect of O_3_ on wet samples and the lower effect on dry samples. Both the lichen and the moss, after the wet exposure, showed a total flattening of the typical OJIP steps, indicating that reduction of plastoquinone A by electrons did not occur, while dry samples showed an intermediate decrease, indicating that the reduction of plastoquinone B by electrons did not occur, confirming the lower susceptibility of the photosynthetic system under the dry state. This behavior was probably determined by the reduced presence of water in these samples (<10%), which may have limited the diffusion of O_3_, since its direct effect on the PSII has been excluded [55]. In fact, when these cryptogamic organisms are dry, most of them put into action strategies to protect their photosynthetic system from photoinactivation [56], which make them less sensitive to pollutants. In addition, the fast disappearance of O_3_, due to its short half-life that ranges from minutes to hours [27], may play an important role.

The response of the antioxidant activity showed slight increases in the dry fumigated samples of *E. prunastri* (+150%) and in the wet ones of *Brachythecium* (+128%). The increases in the antioxidant power after the exposure to O_3_ may be related to the necessity for the organism to maintain a stable balance between ROS production and elimination to preserve its cellular and metabolic integrity. ROS can work as signals to stimulate cellular defenses but, following the exposure to oxidizing compounds (O_3_), their extracellular concentration increases, generating cellular damage. The effects of seasonal O_3_ variations on the antioxidant levels of *Picea rubens*, clones of *Populus* and *Triticum aestivum* were reported [57,58,59], and similarly, fumigations with very low O_3_ concentrations caused increases in all of the indicators of antioxidant activity during the first 20 min of exposure of *Carica papaya* [60]. In addition, prolonged exposure to 50 ppb O_3_ increased the total phenolic content and peroxidase activity of two different wheat cultivars [51], and fumigations with 32 ppb O_3_ for 4 weeks increased the phenolic contents of *Trifolium pratense* cv. Bjursele [61]. The increase in the antioxidant power in dry samples of *E. prunastri* may be, and is probably, related to the ability to counteract the (limited) ROS production, irrespective of an inefficient metabolism. The slight increase in the antioxidant power in the wet samples of *Brachythecium* was probably due to a first step of the metabolism to counteract the deleterious effects of ROS on the photosynthetic machinery, as suggested by the reductions in photosynthetic efficiency.

After one week of recovery from the O_3_ fumigation, dry samples of *E. prunastri* still showed a higher antioxidant power than controls, while wet samples showed a decrease; wet samples of *Brachythecium* showed a higher antioxidant power compared with control samples. The low antioxidant power of wet *E. prunastri* samples may be, and is probably, due to a sort of metabolic stress caused by ROS, as has been observed for higher plants after prolonged exposure to O_3_ [60]. Nevertheless, we may also speculate that the degradation of photobiont cells could have resulted in the formation of molecules with antioxidant properties.

The higher antioxidant expression of wet moss may be due to the already turned on antioxidant metabolism, stimulated to maintain a sort of protection mechanism for the photosynthetic system during its complete recovery [58] or as a consequence of a physiological stress-memory process that protects the organism from subsequent oxidative exposures, as can occur in higher plants following ecological changes [62].

## 5. Conclusions

The fumigation of the lichen *Evernia prunastri* and the moss *Brachythecium* sp. with 1 ppm O_3_ for 1 h highlighted the higher susceptibility of these cryptogamic organisms to this oxidizing pollutant under the hydrated state. An impairment of the photosynthetic efficiency and a reduction in the chlorophyll content were evident. The role of antioxidant power as mechanism of resistance to high O_3_ levels needs to be better clarified with further investigations. Our results confirm that the tolerance to O_3_ of lichens and mosses may be determined by their low water content, which is the case during the peaks of O_3_ occurring during the Mediterranean summer.

## Figures and Tables

**Table 1:** Chlorophyll content (Chl), photosynthetic efficiency (F_V_/F_M_) and antioxidant power % (ARA) in samples of the lichen (L) *Evernia prunastri* and the moss (M) *Brachythecium* sp. exposed dry (D) or wet (W), immediately after (E) 1 h fumigation with 1 ppm O_3_ or after 1 week of recovery (R). Values are expressed as median ± median absolute deviation of ratios to control values. Values in bold indicate significant (*p* < 0.05) differences with control values, capital letters indicate significant (*p* < 0.05) differences between D and W, and lowercase letters indicate significant (*p* < 0.05) differences between E and R. * values were almost zero and were disregarded for the statistical analysis but were considered significantly different from controls and dry samples.

**Parameter**	**LED**	**LEW**	**LRD**	**LRW**
Chl	0.85 ± 0.19 A	0.00B*	0.89 ± 0.47 A	0.00B*
F_V_/F_M_	**0.21 ± 0.09 A**	0.00 B*	**0.19 ± 0.14 A**	0.00B*
ARA	**1.51 ± 0.09 Aa**	1.11 ± 0.08 Ba	**1.19 ± 0.02 Ab**	**0.83 ± 0.01 Bb**
	**MED**	**MEW**	**MRD**	**MRW**
Chl	0.96 ± 0.13 A	**0.48 ± 0.17 Ba**	0.73 ± 0.27	0.73 ± 0.18 b
F_V_/F_M_	**0.35 ± 0.11 Aa**	0.00B*	**0.70 ± 0.14 Ab**	0.00B*
ARA	1.16 ± 0.20 a	**1.30 ± 0.11a**	1.04 ± 0.05 Ab	**1.17 ± 0.01 Bb**
